# The clinical and prognostic value of late Gadolinium enhancement imaging in heart failure with mid-range and preserved ejection fraction

**DOI:** 10.1007/s00380-021-01910-2

**Published:** 2021-07-22

**Authors:** Gijs van Woerden, Dirk J. van Veldhuisen, Thomas M. Gorter, Tineke P. Willems, Vanessa P. M. van Empel, Aniek Peters, Gabija Pundziute, Jeroen W. op den Akker, Michiel Rienstra, B. Daan Westenbrink

**Affiliations:** 1 Department of Cardiology, University of Groningen, University Medical Centre Groningen, Hanzeplein 1, PO Box 30.001, 9700 RB Groningen, The Netherlands; 2 Department of Radiology, University of Groningen, University Medical Centre Groningen, Groningen, The Netherlands; 3 Department of Cardiology, University of Maastricht, Medical University Centre Maastricht, Maastricht, The Netherlands

**Keywords:** HFpEF, HFmrEF, Myocardial scar, Cardiac magnetic resonance imaging, Mortality

## Abstract

Heart failure (HF) with mid-range or preserved ejection fraction (HFmrEF; HFpEF) is a heterogeneous disorder that could benefit from strategies to identify subpopulations at increased risk. We tested the hypothesis that HFmrEF and HFpEF patients with myocardial scars detected with late gadolinium enhancement (LGE) are at increased risk for all-cause mortality. Symptomatic HF patients with left ventricular ejection fraction (LVEF) > 40%, who underwent cardiac magnetic resonance (CMR) imaging were included. The presence of myocardial LGE lesions was visually assessed. T1 mapping was performed to calculate extracellular volume (ECV). Multivariable logistic regression analyses were used to determine associations between clinical characteristics and LGE. Cox regression analyses were used to assess the association between LGE and all-cause mortality. A total of 110 consecutive patients were included (mean age 71 ± 10 years, 49% women, median N-terminal brain natriuretic peptide (NT-proBNP) 1259 pg/ml). LGE lesions were detected in 37 (34%) patients. Previous myocardial infarction and increased LV mass index were strong and independent predictors for the presence of LGE (odds ratio 6.32, 95% confidence interval (CI) 2.07–19.31, *p* = 0.001 and 1.68 (1.03–2.73), *p* = 0.04, respectively). ECV was increased in patients with LGE lesions compared to those without (28.6 vs. 26.6%, *p* = 0.04). The presence of LGE lesions was associated with a fivefold increase in the incidence of all-cause mortality (hazards ratio 5.3, CI 1.5–18.1, *p* = 0.009), independent of age, sex, New York Heart Association (NYHA) functional class, NT-proBNP, LGE mass and LVEF. Myocardial scarring on CMR is associated with increased mortality in HF patients with LVEF > 40% and may aid in selecting a subpopulation at increased risk.

**Supplementary Information:**

The online version contains supplementary material available at 10.1007/s00380-021-01910-2.

## Introduction

Heart failure (HF) with mid-range or with preserved ejection fraction (i.e., left ventricular ejection fraction (LVEF) > 40%) has a high morbidity and mortality rate and there are currently no evidence-based therapies for this condition [1–4]. The lack of therapeutic options for this population is thought to be related to the heterogeneity of the disease [1, 5–7]. Hence, there is an urgent need for identifying more uniform and high-risk subpopulations in HF patients with LVEF > 40% [6]. Although echocardiography is key for the diagnosis and classification of HF with LVEF > 40%, it offers limited opportunities to establish the underlying aetiology [8].

Cardiac magnetic resonance (CMR) imaging offers unique opportunities for tissue characterisation that may assist in the identification of distinct subpopulations of HF with LVEF > 40%. In particular, the ability of CMR to detect focal myocardial scars using late gadolinium enhancement (LGE) imaging is of interest, as myocardial fibrosis is thought to be a pathophysiological hallmark of HF with LVEF > 40% [9]. LGE lesions are associated with poor prognosis in HF with reduced ejection fraction (HFrEF, LVEF ≤ 40%) and the general elderly population without HF, yet surprisingly little is known about the prevalence and prognostic significance of LGE lesions in HF patients with an LVEF > 40% [10, 11]. We therefore sought to determine the prevalence and prognostic value of myocardial LGE lesions in patients with HFmrEF and HFpEF.

## Materials and methods

### Patient population

**Fig. 1:**
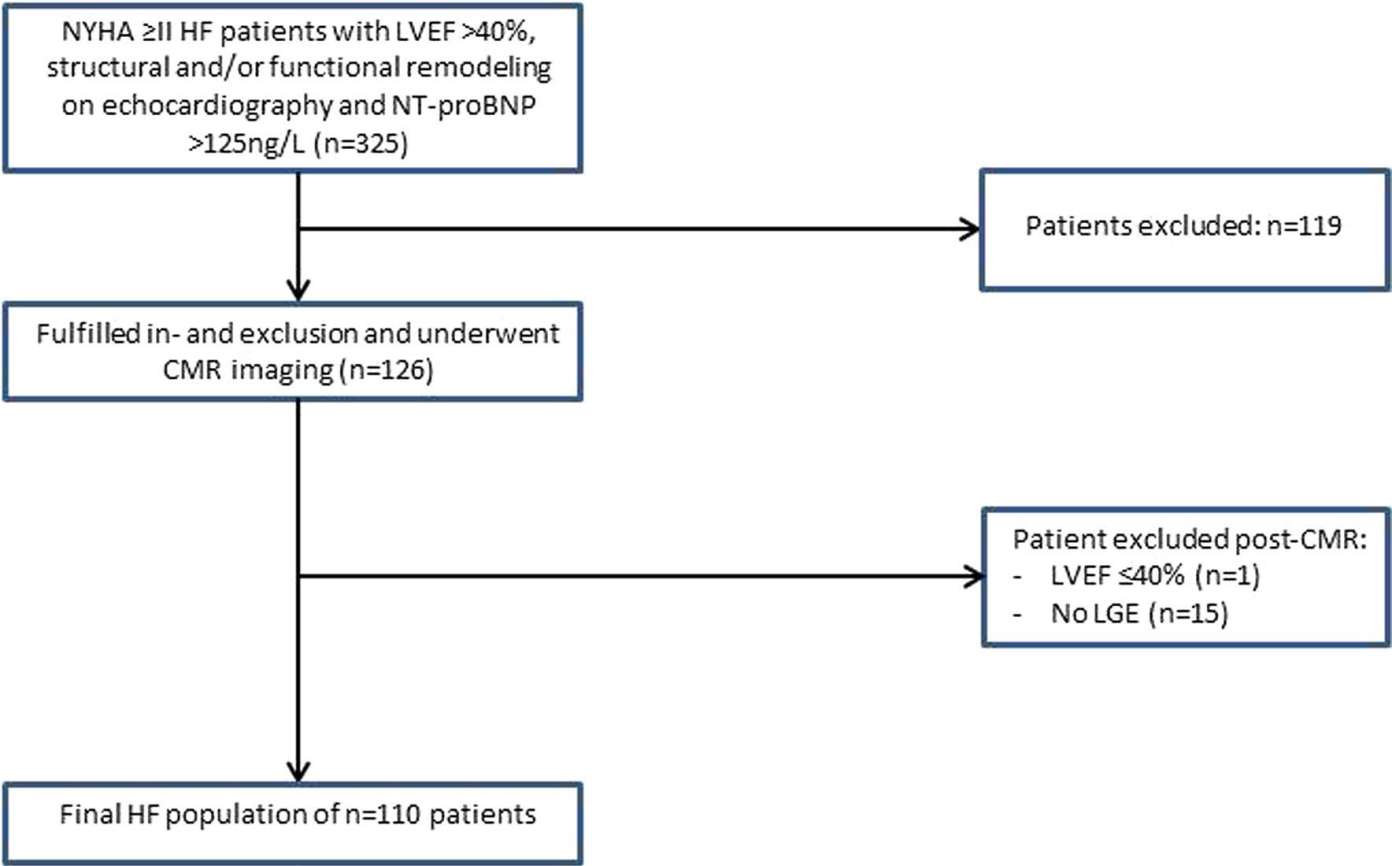
Study flowchart. *NYHA* New York Heart Association, *HF* heart failure, *LVEF* left ventricular ejection fraction, *NT-proBNP* *N*-terminal pro brain natriuretic peptide, *ICD* internal cardiac defibrillator, *CMR* cardiac magnetic resonance, *LGE* late gadolinium enhancement. *No LGE* no gadolinium administration or insufficient quality

Consecutive symptomatic HF patients (New York Heart Association [NYHA] functional class ≥ 2) who had an LVEF > 40% on echocardiography between November 2012 and December 2019 were studied. Patients were eligible if *N*-terminal prohormone of brain natriuretic peptide (NT-proBNP) was > 125 pg/ml and echocardiography showed evidence of LV diastolic dysfunction, left atrial dilatation and/or LV hypertrophy, according to current European Society of Cardiology criteria [5]. Patients were excluded from the present analysis if they had LVEF ≤ 40% on either echocardiography or CMR imaging, (corrected) congenital heart disease, genetically proven hypertrophic-, or dilated cardiomyopathy, or if they had more than moderate left-sided valvular disease. Other exclusion criteria included myocardial infarction < 3 months prior to CMR imaging contra-indications for CMR imaging, including claustrophobia and presence of pacemaker or internal cardiac defibrillator (ICD). A study flowchart is shown in Fig. 1. Previous myocardial infarction was assessed by reviewing the medical records. All patients were part of a standard diagnostic protocol for HF patients with an LVEF > 40%. This protocol consisted of a thorough examination including laboratory testing, echocardiography and routine cardiac magnetic resonance (CMR) imaging for the aetiology of HF. Serum NT-proBNP and estimated glomerular filtration rate (eGFR) were determined using an immunoassay based on electrochemiluminescence (Elecsys, Roche Diagnostics, Mannheim, Germany). The Institutional Review Board of the University Medical Centre Groningen approved the study and because of the retrospective nature of the study, the need for individual informed consent was waived. The present study was in concordance with the principles outlined in the Declaration of Helsinki.

### Echocardiography

Echocardiographic parameters were assessed according to the current recommendations for cardiac chamber quantification and included: LV systolic function, LV diastolic function, left atrial volume index, LV mass index, valvular stenosis and/or regurgitation and the peak pressure gradient across the tricuspid valve [12].

### Cardiac magnetic resonance imaging

#### General assessment

CMR was performed using a standard protocol for the acquisition of cardiac volumes and functional parameters, as previously published by our group [7]. In brief, all CMR studies were performed using a 1.5 Tesla scanner (Philips, Amsterdam, The Netherlands and Siemens, Erlangen, Germany). ECG-triggered cine loop images were obtained during breath hold at end-expiration, using a retrospectively gated cine steady-state, free-precession sequence. Approximately, 15 short-axis slices from base to apex were obtained. CMR images for LV, right ventricular (RV), left atrial and right atrial volume, function and contractility were analysed off-line by two observers (G.W. and T.M.G.) using dedicated software (QMass 7.6, QStrain 2.0, Medis, Leiden, The Netherlands). The assessment of cardiac volumes, function, and contractility have previously been described by our group [7]. HF patients with LVEF of 40–49% were classified as HFmrEF, and HF patients with LVEF of ≥ 50% as HFpEF, according to current ESC criteria [5].

#### Late gadolinium enhancement

Using the same slice locations, LGE images were acquired 10 min after intravenous administration of 0.2 mmol/kg gadolinium-based contrast agent (Dotarem, Gorinchem, the Netherlands; 0.2 mmol/kg and Gadovist, Berlin, Germany; 0.2 mmol/kg) [13, 14]. Typical sequence parameters were: (Siemens: TE 1.08 ms; TR 700 ms; flip angle 40°; voxel size 2.3 × 2.3 × 6.0 mm, Philips: TE 0.93 ms; TR 2.1 ms; flip angle 25°; voxel size 1.6 × 1.8 × 8.0 mm.) The presence of LGE was visually evaluated in a blinded fashion by two observers (G.W. and T.M.G) and confirmed by an observer experienced in clinical cardiac magnetic resonance imaging (B.D.W). The full-width half-maximum technique was used to quantify LGE lesions [15].

#### T1 mapping

Native and post-contrast T1 measurements were performed using a Modified Look-Locker inversion recovery (MOLLI) sequence (Siemens: TE 1.13 ms; TR 303.92 ms; flip angle 35°; matrix 360 × 360 mm, voxel size 1.40 × 1.40 × 8 mm, Philips: TE 0.93 ms; TR 2.1 ms; flip angle 35°; matrix 300 × 300 mm, voxel size 2.0 × 2.0 × 10 mm.) Native and post-contrast T1 imaging was performed before and at least 10 min after administration of the gadolinium-based contrast agent. T1 measurements were performed on the mid-papillary short-axis slice. For T1 measurements, the endo- and epicardial borders of the LV were manually traced by three observers (G.W., T.M.G. and A.P.) using QMass 7.6 and 8.1 (Medis, Leiden, The Netherlands). When measuring T1 times of the myocardium, extra attention was paid to exclude the blood volume and regions of LGE lesion. Extracellular volume (ECV) fraction was calculated from the native- and post-contrast T1 times and blood haematocrit.

### Outcome

The endpoint used in this study was all-cause mortality. Follow-up time was defined as the time from CMR to death. Medical records were reviewed to assess whether the endpoint was met.

### Statistical analysis

Data are presented as numbers and percentages, mean ± standard deviation or median with interquartile ranges. Differences in categorical variables were analysed using the Chi-squared test or Fisher’s exact test, where appropriate. Differences in normally distributed continuous variables were compared using the independent-sample t test or Wilcoxon signed rank test if variables were not normally distributed. Univariable logistic regression was used to determine which covariates were associated with the presence of LGE lesions. To minimize the risk of type II errors, we considered *p* < 0.1 as a significant univariable association and these variables were entered into a multivariable backward selection model. First-line interaction between all covariates with *p* < 0.1 for the association with the presence of LGE was assessed.

A Kaplan–Meier plot with log rank test was used to display relation between LGE and outcome, which was defined as all-cause mortality. The association between LGE and all-cause mortality was assessed using a univariable Cox proportional hazard regression model. The multivariable Cox regression model was adjusted for age, sex, NYHA functional class, NT-proBNP, LGE mass and LVEF as these variables are strong determinants of outcome in patients with HFpEF [16–18]. The Cox proportional hazards assumption was assessed by visually inspecting the log minus log survival plots over time, which showed proportionality. Statistical analyses were performed using SPSS (Version 23, Chicago, Illinois). Statistical significance was considered achieved at a *p* value < 0.05.

## Results

### Clinical characteristics

The study population consisted of 110 HF patients (Fig. 1). In 15 patients, LGE imaging was either not performed or the scan was of insufficient quality to assess LGE and these patients were excluded. Mean age was 71 ± 10 years, 49% were female and the median NT-proBNP was 1259 pg/ml (Table 1). The clinical characteristics stratified by HFpEF or HFmrEF are depicted in Supplementary Table 1. HFpEF patients were more often female (59 vs. 30%, *p* = 0.004), and more often had a history of hypertension (82 vs. 60%, *p* = 0.01) and less often renal dysfunction (30 vs. 51%, *p* = 0.03).

**Table 1:** Patient characteristics

	Total (*n* = 110)	LGE- (*n* = 73)	LGE + (*n* = 37)	*p*
Demographics				
Age (years)	70.8 ± 9.8	71.1 ± 10.0	70.1 ± 9.4	0.6
BMI, (kg/m^2^)	29.5 ± 5.9	30.0 ± 6.3	28.5 ± 4.9	0.2
Male sex, *n* (%)	56 (51%)	28 (38%)	28 (76%)	**< 0.001**
NYHA class,* n* (%)				0.4
II	65 (59%)	41 (56%)	24 (65%)	
III	45 (41%)	32 (44%)	13(35%)	
Systolic blood pressure (mmHg)	142.3 ± 20.8	145.2 ± 18.0	136.1 ± 25.0	0.1
Diastolic blood pressure (mmHg)	74.7 ± 14.5	73.5 ± 14.4	77.2 ± 14.5	0.2
Heart rate (bpm)	71.8 ± 13.2	72.6 ± 13.9	70.2 ± 11.8	0.4
Coronary revascularization^a^ (%)	32 (29%)	15 (21%)	17 (46%)	**0.006**
Comorbidities, *n* (%)				
Hypertension	82 (75%)	55 (75%)	27 (73%)	0.8
Diabetes mellitus	35 (32%)	21 (29%)	14 (38%)	0.3
Renal dysfunction	41 (37%)	28 (38%)	13 (35%)	0.7
Myocardial infarction	26 (24%)	8 (11%)	18 (49%)	**< 0.001**
Coronary artery disease^b^	37 (34%)	16 (22%)	21 (57%)	**< 0.001**
Atrial fibrillation	52 (47%)	36 (49%)	16 (43%)	0.5
Medications, *n* (%)				
Beta blocker	98 (89%)	65 (89%)	33 (89%)	1.0
ACEi/ARB	72 (66%)	45 (62%)	27 (73%)	0.2
Mineral receptor antagonist	44 (40%)	31 (43%)	13 (35%)	0.5
Diuretic	99 (90%)	66 (90%)	33 (89%)	0.8
Laboratory testing				
NT-proBNP (pg/ml)	1259 (670–2531)	1202 (630–2242)	1726 (688–3631)	0.1
TroponinT (ng/L)	20 (13–31)	17 (10–27)	27 (16–35)	**0.007**
eGFR (ml/min*1.73m^2^)	57 (42–77)	57 (41–76)	58 (44–81)	0.8
Echocardiography				
LV GLS	12.1 ± 3.8	12.3 ± 4.2	11.6 ± 2.7	0.3
E/e'	11 (8–15)	11 (8–15)	11 (9–16)	0.6
LVMI (g/m^2^)	107.1 ± 40.0	102.8 ± 36.7	115.2 ± 45.0	0.1
LAVI (ml/m^2^)	49.6 ± 22.4	50.5 ± 23.6	47.8 ± 20.0	0.6

Statistical significance was considered achieved at a *p* value < 0.05

Quantitative data are presented as mean ± standard deviation or median with interquartile ranges. Qualitative data are presented as *n* (%). *p *value comparing heart failure (HF) with late gadolinium enhancement (LGE) and HF without LGE. *ACEi* angiotensin converting enzyme inhibitor, *ARB* angiotensin II receptor blocker, *BMI* body mass index, *LV GLS* left ventricular global longitudinal strain, *LVMI* left ventricular mass index, *LAVI* left atrial volume index, *NYHA* New York Heart Association

^a^Revascularization was defined as follows: underwent percutaneous coronary intervention and/or coronary artery bypass grafting

^b^Coronary artery disease was defined as follows: history of myocardial infarction, percutaneous intervention and/or coronary artery bypass grafting

### Clinical and CMR characteristics in patients with or without LGE lesions

**Fig. 2:**
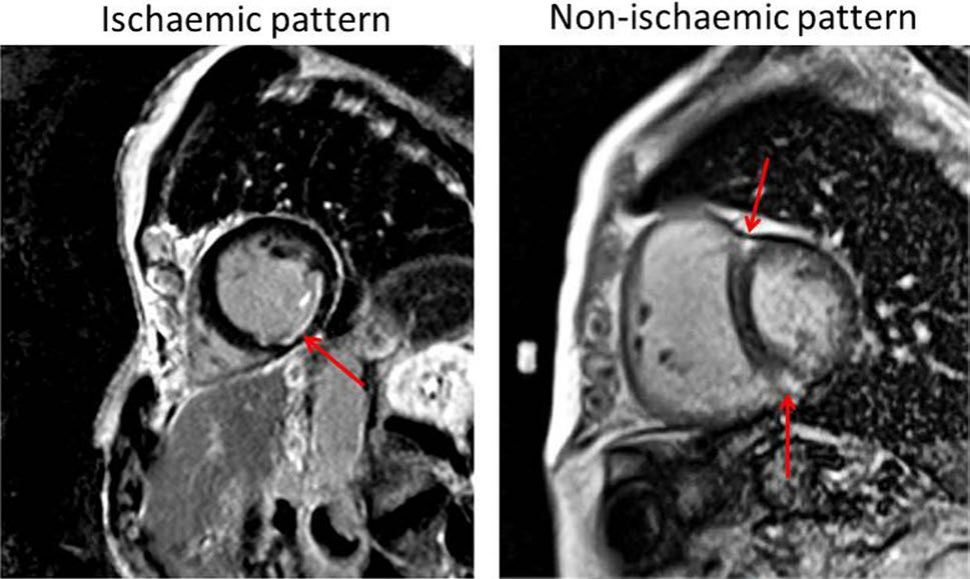
Examples of late gadolinium enhancement in heart failure patients with left ventricular ejection fraction > 40%

LGE lesions were observed in 37 (34%) patients, of which 22 (20%) were ischemic-, and 15 (14%) were considered to be non-ischemic LGE lesions (Fig. 2). Of the 22 patients with an ischemic LGE lesion, 8 (36%) did not have a history of myocardial infarction.

Patients with an LGE lesion were more often male (76 vs. 38%, *p* < 0.001) and had a previous myocardial infarction more frequently (49 vs. 11%, *p* < 0.001). In addition, patients with an LGE lesion had increased levels of Troponin T compared to those without LGE (27 vs. 17 ng/L, *p* = 0.007). The median time interval between troponin T assessment and CMR imaging was 37 [16–77] days. CMR measurements are depicted in Table 2. LV mass index was almost 20% higher in patients with LGE lesions compared to patients without LGE (*p* = 0.02). LVEF on echocardiography and CMR were moderately associated (*r* = 0.38, *p* < 0.001). CMR characteristics for HFpEF and HFmrEF are displayed in Supplementary Table 2.

**Table 2:** CMR characteristics: patients with and without late gadolinium enhancement lesions

	HF LGE- (*n* = 73)	HF LGE + (*n* = 37)	*p*
Structure and function			
LVESVI (ml/m^2^)	41.0 ± 16.3	47.2 ± 18.9	0.08
LVEDVI (ml/m^2^)	88.1 ± 24.7	95.4 ± 25.7	0.2
LVEF (%)	54.6 ± 7.8	51.7 ± 9.1	0.09
LVMI (g/m^2^)	55.2 ± 19.3	65.3 ± 25.4	**0.02**
LV GLS (%)	17.7 ± 5.1	16.8 ± 4.3	0.4
LV GCS (%)	22.8 ± 6.4	21.0 ± 6.0	0.2
LV torsion (°)	6.7 (2.3–14.1)	8.3 (4.3–20.1)	0.07
RVESVI (ml/m^2^)	39.4 ± 14.4	42.4 ± 21.0	0.4
RVEDVI (ml/m^2^)	83.9 ± 21.2	86.2 ± 25.7	0.6
RVEF (%)	53.7 ± 9.0	52.2 ± 11.6	0.5
RV GLS (%)	20.0 ± 5.7	20.7 ± 7.3	0.6
LAESVI (ml/m^2^)	60.2 ± 22.4	60.9 ± 22.6	0.9
RAESVI (ml/m^2^)	46.0 ± 22.6	45.9 ± 12.3	1.0
Late gadolinium enhancement			
LGE, % of LV mass		6.6 (4.4–11.2)	
T1 mapping (*n* = 75)			
Native myocardial T1 (ms)	1013 ± 50	1043 ± 39	**0.01**
Post-contrast myocardial T1 (ms)	424 ± 42	413 ± 39	0.3
ECV (%)	26.6 ± 3.3	28.6 ± 3.7	**0.04**

Statistical significance was considered achieved at a *p* value < 0.05

Data are presented as mean ± standard deviation or median with interquartile ranges. *p* value comparing heart failure (HF) with late gadolinium enhancement (LGE) and HF without LGE. *ECV* extracellular volume, *LVEF* left ventricular ejection fraction, *LVEDVI* left ventricle end-diastolic volume index, *LVESVI* left ventricle end-systolic volume index, *LV GLS* left ventricular global longitudinal strain, *LV GCS* left ventricular global circumferential strain, *LVMI* left ventricle mass index, *RVEF* right ventricular ejection fraction, *RVEDVI* right ventricle end-diastolic volume index, *RVESVI* right ventricle end-systolic volume index, *RV GLS* right ventricular global longitudinal strain, *LAESVI* left atrial end-systolic volume index, *RAESVI* right atrial end-systolic volume index

### T1 mapping and ECV

T1 mapping was performed in 75 (68%) patients. In patients with LGE lesions, native T1 times and ECV were significantly increased compared to patients without LGE lesions (1043 vs. 1013 ms, *p* = 0.01 and 28.6 vs. 26.6%, *p* = 0.04, respectively (Table 2).

### Associations between clinical and CMR characteristics and LGE lesions

Patient- and CMR characteristics with a univariable association (*p* < 0.1) with LGE lesions are depicted in Table 3. Previous myocardial infarction and LV mass index were strong predictors for the presence of LGE lesions and remained independently associated after adjustment (Odds ratio 6.32 with 95% confidence interval 2.07–19.31, *p* = 0.001 and 1.68 (1.03–2.73), *p* = 0.04, respectively). Forced entry of age and sex into the model did not improve the predictive accuracy of the model.

**Table 3:** Clinical and CMR determinants associated with the presence of late gadolinium enhancement

	OR	Univariate			Multivariate	
		95% CI	*p*	OR	95% CI	*p*
Demographics						
Male sex	5.00	2.06–12.14	< 0.001	3.65	1.32–10.10	0.01
Systolic blood pressure	0.98	0.96–0.99	0.04	0.97	0.95–0.99	0.02
Coronary revascularization	3.29	1.39–7.77	0.007			
Comorbidities						
Myocardial infarction	7.70	2.90–20.45	< 0.001	6.32	2.07–19.31	0.001
Coronary artery disease	4.68	1.99–10.99	< 0.001			
Laboratory testing						
TroponinT	2.03	1.14–3.64	0.02			
Cardiac magnetic resonance						
LVESVI	1.02	0.99–1.04	0.08			
LVEF	0.96	0.91–1.00	0.09			
LVMI^a^	1.60	1.05–2.44	0.03	1.68	1.03–2.73	0.04

*LVESVI* left ventricular end-systolic volume index, *LVEF* left ventricular ejection fraction, *LVMI* left ventricle mass index

^a^Per standard deviation increase

### Association between myocardial scar and outcome

**Fig. 3:**
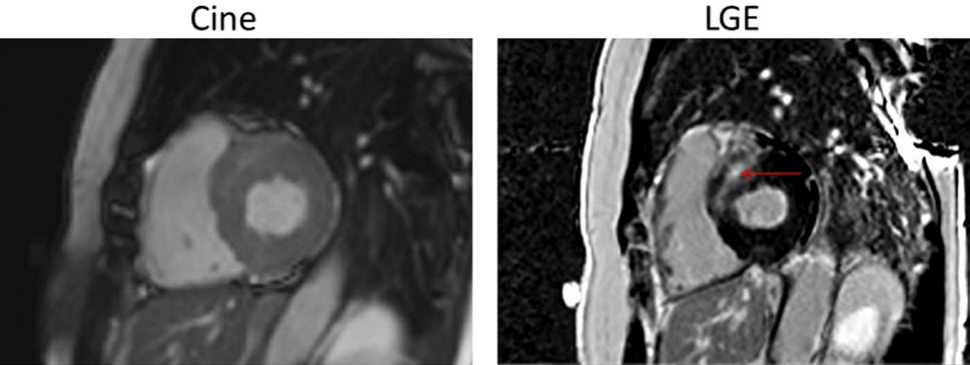
Typical example of a patient who died due to cardiovascular causes with a late gadolinium enhancement lesion on cardiac magnetic resonance imaging

**Fig. 4:**
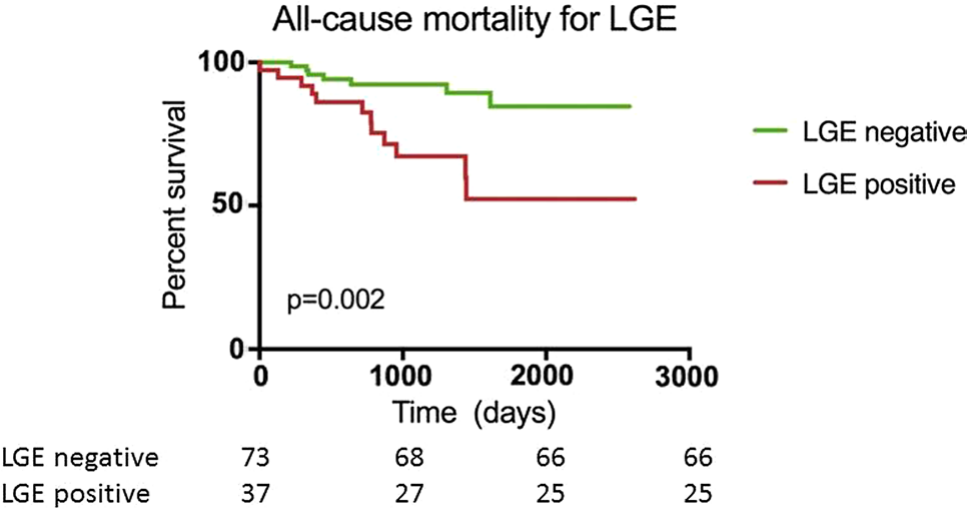
Kaplan–Meier curves of heart failure patients stratified for the presence of late gadolinium enhancement. *LGE* = late gadolinium enhancement

During a median follow-up of 34 months (interquartile range 19–53), 19 (17%) patients died. Of these 19 patients, 11 died due to cardiovascular causes and 8 due to a variety of non-cardiovascular causes such as cancer, and chronic obstructive pulmonary disease. In Fig. 3, a typical CMR example is shown of a patient with a non-ischemic LGE lesion, who died due to cardiovascular causes. Mortality was significantly higher in patients with LGE lesions (Log Rank *p* = 0.002, Fig. 4) The presence of LGE was an independent determinant of all-cause mortality after the adjustment for age, sex, NYHA functional class, NT-proBNP, LGE mass and LVEF (adjusted hazards ratio 5.3, 95% confidence interval 1.5–18.1, *p* = 0.009) (Table 4). LV diastolic dysfunction, LV hypertrophy and left atrial volume index on echocardiography were all not associated with mortality (data not shown).

**Table 4:** Cox regression analysis for all-cause mortality

	HR	Univariate		HR	Multivariate	
		95% CI	*p*		95% CI	*p*
LGE presence (yes vs. no)	3.9	1.5–9.9	**0.005**	5.3	1.5–18.1	**0.009**
LGE mass (per 5%)	1.00	0.96–1.1	0.8	0.9	0.8–1.1	0.4
Age (per 5 years)	1.05	0.97–1.1	0.3	1.08	0.97–1.2	0.2
Sex (male vs. female)	1.5	0.6–3.9	0.4	1.3	0.4–4.4	0.7
NYHA functional class (III vs. II)	1.9	0.7–4.8	0.2	1.8	0.7–4.9	0.2
NT-proBNP (per doubling)	1.3	0.9–1.8	0.2	1.0	1.0–1.0	0.6
LVEF (per 5%)	1.00	0.9–1.1	0.9	0.9	0.8–1.1	0.9

Statistical significance was considered achieved at a *p* value < 0.05

*LGE* late gadolinium enhancement, *NYHA* New York Heart Association, *LVEF* left ventricular ejection fraction, *HR* hazards ratio, *CI* confidence interval

## Discussion

In the present study, we sought to determine whether CMR with LGE imaging could aid in the identification of a high-risk subgroup of patients with well-defined HFmrEF and HFpEF. Myocardial scarring was present in one third of patients with HFmrEF and HFpEF. In addition, myocardial scars were associated with left ventricular hypertrophy and a history of myocardial infarction. In patients with myocardial scar, ECV values were significantly increased in the myocardium remote from the LGE lesion. Lastly, the presence of myocardial scar was associated with poor outcome. Together, our findings indicate that CMR with LGE imaging may aid in detecting a subpopulation of high-risk HF patients with LVEF > 40% and its use should, therefore, be considered in the work-up of HF.

The identification of high-risk subgroups in HF with LVEF > 40% could be a first step towards better, more tailored treatment of this disease. Our data suggest that the detection of myocardial scar with CMR can help identify such a high-risk subgroup. It is plausible that the difference in survival between patients with- and without an LGE lesion is due to an increase in arrhythmic death. Earlier studies in HFrEF patients showed that the presence of an LGE lesion was associated with ventricular arrhythmic events [19]. However, a recent study by our group showed that sustained- and non-sustained ventricular tachyarrhythmias are uncommon in patients with HFmrEF and HFpEF [20]. Therefore, the association between LGE and ventricular arrhythmias seen in HFrEF, may be different for HFmrEF and HFpEF. In contrast, no distinct subgroups could be made based on cardiac remodelling on echocardiography, further supporting the use of CMR in the work-up of patients with HFmrEF and HFpEF.

Our data support the findings of a prior study, which reported increased mortality and HF hospitalizations in HFpEF patients with myocardial scar on CMR [21]. However, two different studies in HFpEF patients failed to detect a clear association between LGE lesions and outcome [22, 23]. A potential explanation for this discrepancy may be that the follow-up period of the current study is longer than these studies (median of almost 3 years vs. 1 and 2 years). In addition, we also included HF patients with LVEF between 41 and 49%, which is different from previous reports who solely included HF patients with LVEF > 50%.

In our study, LVEF and LV GLS were not associated with LGE or all-cause mortality. This is in line with previous studies, showing that LV systolic function is not independently associated with outcome in patients with HFpEF [24]. LGE volume has been associated with deterioration of LVEF in dilated cardiomyopathy patients in follow-up [25]. Whether this also true for patients with HFmrEF and HFpEF remains to be elucidated.

Native T1 and ECV values were significantly increased in the myocardium remote from the LGE lesion, indicating that patients with a focal myocardial scar also display more interstitial myocardial fibrosis. These findings suggest that focal fibrosis is also associated with more general fibrotic heart disease in HF with LVEF > 40% and may reflect a higher burden of heart failure.

### Study limitations

Our study is exploratory in nature and suffers from general limitations associated with its retrospective nature. In addition, a few specific limitations should be acknowledged. First, because T1/ECV mapping became available in our center during the course of the study, it is only available in 75 out of 110 patients. Second, although our population was meticulously characterised, our sample size is relatively small and our mortality rate was lower than in most HFmrEF/HFpEF trials. The incidence and prognostic significance of LGE may, therefore, be different in the general HFmrEF/HFpEF population. Fourth, we did not include a control group, and we cannot rule out that the association between LGE and mortality also applies to similar subjects without HF.

### Clinical implications

Our study has shown that a considerable amount of HF patients with LVEF > 40% have myocardial scars and these patients are at increased risk. The presence of an LGE lesion may not only aid in follow-up, but the pattern of LGE may also assist in the diagnosis of the underlying cause of HFpEF/HFmrEF. For instance, of the 22 patients in our study with an ischemic LGE scar, 8 (36%) did not have a history of myocardial infarction. These findings suggest that HF was caused by a silent myocardial infarction, and these patients may benefit from anti-ischemic therapy. On the other hand, clinical trials may also profit from selecting HF patients with LGE lesions, as this would result in a study population at increased risk.

## Conclusions

Myocardial scarring on CMR is associated with increased mortality in HF patients with LVEF > 40% and may aid in selecting a subpopulation of patients at increased risk.

## Supplementary Information

Below is the link to the electronic supplementary material.

Supplementary file1 (DOCX 19 KB)
