# Supplementary material for: The clinical and prognostic value of late Gadolinium enhancement imaging in heart failure with mid-range and preserved ejection fraction
Source: Heart Vessels. 2021 Jul 22;37(2):273–81. doi: 10.1007/s00380-021-01910-2 (PMC8794962; doi:10.1007/s00380-021-01910-2)
Supplement: Supplementary file 1 — Supplementary file1 (DOCX 19 KB) [file 380_2021_1910_MOESM1_ESM.docx]

**Supplementary Table 1** Clinical characteristics based on HFmrEF and HFpEF

|  | **HFmrEF (n=37)** | **HFpEF (n=73)** | **P** |
| --- | --- | --- | --- |
| ***Demographics*** |  |  |  |
| Age (years) | 68.9 ± 10.1 | 71.7 ± 9.5 | 0.2 |
| BMI, (kg/m^2)^ | 28.4 ± 5.6 | 30.0 ± 6.0 | 0.2 |
| Male sex, n (%) | 26 (70%) | 30 (41%) | **0.004** |
| *NYHA class, n(%)* |  |  | 0.9 |
| II | 23 (62%) | 42 (58%) |  |
| III | 14 (38%) | 31 (43%) |  |
| Systolic blood pressure (mmHg) | 138.4± 23.3 | 144.3 ± 19.3 | 0.2 |
| Diastolic blood pressure (mmHg) | 74.6 ± 16.1 | 74.7 ± 13.6 | 0.9 |
| Heart rate (bpm) | 72.9 ± 12.8 | 71.2 ± 13.5 | 0.5 |
| Coronary revascularization* (%) | 14 (38%) | 18 (25%) | 0.2 |
| ***Comorbidities, n(%)*** |  |  |  |
| Hypertension | 22 (60%) | 60 (82%) | **0.01** |
| Diabetes mellitus | 13 (35%) | 22 (30%) | 0.2 |
| Renal dysfunction | 19 (51%) | 22 (30%) | **0.03** |
| Myocardial infarction | 10 (27%) | 16 (22%) | 0.6 |
| Coronary artery disease** | 16 (43%) | 21 (29%) | 0.1 |
| Atrial fibrillation | 16 (43%) | 36 (49%) | 0.5 |
| ***Medications, n(%)*** |  |  |  |
| Beta blocker | 34 (92%) | 64 (88%) | 0.5 |
| ACEi/ARB | 26 (70%) | 46 (63%) | 0.5 |
| Mineral receptor antagonist | 14 (38%) | 30 (41%) | 0.7 |
| Diuretic | 35(95%) | 64 (88%) | 0.3 |
| ***Laboratory testing*** |  |  |  |
| NT-proBNP (pg/ml) | 1655 [767 - 3147 | 1137 [630 - 2254] | 0.1 |
| TroponinT (ng/L) | 24 [12 - 32] | 19 [13 - 31] | 0.5 |
| eGFR (ml/min*1.73m2) | 53 [42 - 76] | 58 [43 - 78] | 0.4 |
| ***Echocardiography*** |  |  |  |
| LV GLS | 11.0 ± 2.4 | 12.6 ± 4.2 | 0.03 |
| E/e' | 9.2 [8.2 - 13.9] | 12.7 [9.0 - 17.0] | 0.1 |
| LVMI (g/m2) | 116.1 ± 37.7 | 102.4 ± 40.6 | 0.1 |
| LAVI (ml/m^2^) | 48.4 ± 21.7 | 50.2 ± 22.9 | 0.7 |

Quantitative data are presented as mean ± standard deviation or median with interquartile ranges. Qualitative data are presented as *n* (%). P*-*value comparing heart failure (HF) with mid-range ejection fraction (HFmrEF) and preserved ejection fraction (HFpEF). ACEi=angiotensin converting enzyme inhibitor, ARB=angiotensin II receptor blocker, BMI=body mass index, LVEF=left ventricular ejection fraction, LVMI=left ventricular mass index, LAVI=left atrial volume index, NYHA=New York Heart Association.

* Revascularization was defined as follows: underwent percutaneous coronary intervention and/or coronary artery bypass grafting

** Coronary artery disease was defined as follows: history of myocardial infarction, percutaneous intervention and/or coronary artery bypass grafting

**Supplementary Table 2** CMR characteristics stratified on HFmrEF and HFpEF

|  | **HFmrEF (n=37)** | **HFpEF (n=73)** | **P** |
| --- | --- | --- | --- |
| ***Volumes, function and mass*** |  |  |  |
| LVESVI (ml/m2) | 58.5 ± 16.0 | 35.2 ± 12.0 | **<0.001** |
| LVEDVI (ml/m^2^) | 105.7 ± 25.8 | 82.9 ± 21.2 | **<0.001** |
| LVEF (%) | 44.9 ± 3.2 | 58.0 ± 6.4 | **<0.001** |
| LVMI (g/m2) | 62.4 ± 22.1 | 56.7 ± 21.8 | 0.2 |
| LV GLS (%) | 14.1 ± 4.3 | 19.1 ± 4.2 | **<0.001** |
| LV GCS (%) | 17.4 ± 4.4 | 24.6 ± 5.7 | **<0.001** |
| LV Torsion (°) | 5.5 [2.5 - 14.0] | 8.6 [4.0 - 16.0] | 0.2 |
| RVESVI (ml/m^2^) | 44.3 ± 17.5 | 38.5 ± 16.4 | 0.1 |
| RVEDVI (ml/m^2^) | 86.7 ± 22.8 | 83.7 ± 22.8 | 0.5 |
| RVEF (%) | 49.9 ± 10.1 | 54.8 ± 9.5 | **0.02** |
| RV GLS (%) | 18.0 ± 5.5 | 21.4 ± 6.3 | **0.007** |
| LAESVI (ml/m^2^) | 61.7 ± 24.6 | 59.8 ± 20.3 | 0.6 |
| RAESVI (ml/m^2^) | 48.5 ± 25.7 | 44.6 ± 20.6 | 0.4 |
| ***Late gadolinium enhancement*** |  |  |  |
| LGE present | 16 (43%) | 21 (29%) | 0.1 |
| LGE, % of LV mass | 0 [0.0 - 9.2] | 0 [0.0 - 0.5] | **0.03** |
| ***T1 mapping (n=75)*** |  |  |  |
| Native myocardial T1 (ms) | 1030 ± 53 | 1019 ± 46 | 0.4 |
| Post-contrast myocardial T1 (ms) | 409 ± 44 | 426 ± 39 | 0.1 |
| ECV (%) | 28.1 ± 4.0 | 26.9 ± 3.2 | 0.2 |

Data are presented as mean ± standard deviation or median with interquartile ranges. P*-*value comparing heart failure (HF) with mid-range ejection fraction (HFmrEF) and preserved ejection fraction (HFpEF). ECV=extracellular volume, LGE=late gadolinium enhancement, LVEF=left ventricular ejection fraction, LVEDVI=left ventricle end-diastolic volume index, LVESVI=left ventricle end-systolic volume index, LVMI=left ventricle mass index, LVCI=left ventricle cardiac index, RVEF=right ventricular ejection fraction, RVEDVI=right ventricle end-diastolic volume index, RVESVI=right ventricle end-systolic volume index, LAESVI=left atrial end-systolic volume index, RAESVI=right atrial end-systolic volume index
